# Butyrate Increases Intracellular Calcium Levels and Enhances Growth Hormone Release from Rat Anterior Pituitary Cells via the G-Protein-Coupled Receptors GPR41 and 43

**DOI:** 10.1371/journal.pone.0107388

**Published:** 2014-10-13

**Authors:** Maria Consolata Miletta, Vibor Petkovic, Andrée Eblé, Roland A. Ammann, Christa E. Flück, Primus-E. Mullis

**Affiliations:** 1 Division of Paediatric Endocrinology, Diabetology and Metabolism and Department of Clinical Research, University Children's Hospital, Bern, Switzerland; 2 Department of Paediatrics, University of Bern, Bern, Switzerland; National Institute of Agronomic Research, France

## Abstract

Butyrate is a short-chain fatty acid (SCFA) closely related to the ketone body ß-hydroxybutyrate (BHB), which is considered to be the major energy substrate during prolonged exercise or starvation. During fasting, serum growth hormone (GH) rises concomitantly with the accumulation of BHB and butyrate. Interactions between GH, ketone bodies and SCFA during the metabolic adaptation to fasting have been poorly investigated to date. In this study, we examined the effect of butyrate, an endogenous agonist for the two G-protein-coupled receptors (GPCR), GPR41 and 43, on non-stimulated and GH-releasing hormone (GHRH)-stimulated hGH secretion. Furthermore, we investigated the potential role of GPR41 and 43 on the generation of butyrate-induced intracellular Ca^2+^ signal and its ultimate impact on hGH secretion. To study this, *wt*-hGH was transfected into a rat pituitary tumour cell line stably expressing the human GHRH receptor. Treatment with butyrate promoted hGH synthesis and improved basal and GHRH-induced hGH-secretion. By acting through GPR41 and 43, butyrate enhanced intracellular free cytosolic Ca^2+^. Gene-specific silencing of these receptors led to a partial inhibition of the butyrate-induced intracellular Ca^2+^ rise resulting in a decrease of hGH secretion. This study suggests that butyrate is a metabolic intermediary, which contributes to the secretion and, therefore, to the metabolic actions of GH during fasting.

## Introduction

Growth hormone (GH) is a member of the somatotropin/prolactin family of hormones and it is secreted in a pulsatile manner by the pituitary gland. Beyond its well-known effects on longitudinal growth during childhood and adolescence, GH plays a crucial role in controlling energy homeostasis, particularly during energy restriction and fasting [1]. By increasing lipolysis and protein retention, GH impairs suppression of hepatic glucose production and decreases insulin-dependent glucose disposal [2]–[4]. However, potential secondary mediators that contribute to the metabolic action of GH during fasting have not been investigated in great detail.

Butyrate is a short-chain fatty acid (SCFA) produced by bacterial anaerobic fermentation in the gut and is subsequently released into the bloodstream. It is structurally and functionally related to the ketone body ß-hydroxybutyrate (BHB) [5], the major source of energy during prolonged exercise and starvation [6]and is an endogenous agonist for the two G-protein-coupled receptors (GPCR), GPR41 and 43 [7]. During fasting when the liver switches to fatty acid oxidation, a rise in serum GH is observed together with the accumulation of BHB and SCFA such as acetate, propionate and butyrate. The metabolic and hormonal mechanisms by which nutritional deprivation affects the hypothalamic–somatotrophic axis are not completely understood. Until recently, the regulation of GH release was believed to represent the net result of the antagonistic actions of hypothalamic growth hormone releasing hormone (GHRH) and somatostatin (SRIF) on the pituitary, as well as negative feedback via circulating insulin-like growth factor I (IGF-I) [8]. The effect of butyrate and BHB on GH secretion is poorly investigated and it remains unclear whether butyrate induces GH secretion by a direct action on somatotroph cells of the pituitary gland.

Butyrate exerts its action by binding to the receptors GPR41 and GPR43 [7], [9], the two putative GPCR for SCFA sharing 40% of their amino acid sequence, which has been preserved across several mammalian species [7], [9], [10]. Both receptors respond to SCFAs containing two to five carbons, although a preference of GPR43 for C3–C5 fatty acid and of GPR41 for C2 and C3 chain lengths have been reported [7], [9], [11]. The receptors differ in their intracellular signalling capabilities, with GPR43 coupling to either G_q_ or G_i/o_ and GPR41 exclusively activating G_i/o_ pathway [7], [9], [11]. The finding that both receptors are located in the rat pituitary [12] suggests that butyrate may use this pathway to modulate GH secretion.

Therefore, the aim of this study was to examine the effect of butyrate on hGH production and secretion under non-stimulated and GHRH-stimulated conditions. Moreover, using a heterologous cell system based on GC cells (rat pituitary tumour cell line) stably expressing the hGHRH-receptor (GC-GHRHR cells) which were transiently transfected with *wt*-hGH, we investigated the potential role of GPR41 and 43 in butyrate-induced Ca^2+^ signalling and its possible impact on hGH secretion.

## Materials and Methods

### 2.1. Cell culture and treatment

Rat pituitary cell line, GC cells [13], stably transfected with hGHRHR (GC-GHRHR) were cultured in DMEM (4.5 g/liter glucose) supplemented with 15% heat-inactivated horse serum, 2.5% heat-inactivated fetal calf serum, 10 mM Na-Pyruvate (LifeTechnologies, Invitrogen AG, Basel, Switzerland), and 100 U/liter penicillin/streptomycin.

### 2.2. Expression vectors and transfection

Human wild-type GH (*wt*-hGH) was cloned in pXGH5 (Nichols Institute Diagnostics, San Clemente, CA) as previously described [14]. GC-GHRHR were transiently transfected with *wt*-hGH, using Amaxa nucleofection (Lonza Group, Switzerland), with nucleofector solution L using program T-05. As a negative control, GC-GHRHR cells were also transfected with an empty vector (puc18). The transfection efficiency was checked using EGFP-N1 (enhanced green fluorescent plasmid) and results (cells transfection efficiency was between 65–70%) were found to be consistent throughout independent experiments.

### 2.3. GHRH stimulation and butyrate treatment

Six hours after transfection, GC-GHRHR cells were washed in PBS and the culture medium was changed to OPTIMEM medium (Life Technologies, Invitrogen AG). Thereafter, the cells were either stimulated or not with 10 nM GHRH (Bachem AG, Bubendorf, Switzerland) and treated or not with 5 mM butyrate (added as a sodium butyrate) (Sigma Aldrich, Poole, UK) for 24 h. In addition, as a negative control, GC-GHRHR cells were transfected with an empty vector.

### 2.4. RNA isolation and RT-PCR

Total RNA was extracted from GC-GHRHR cells 24 h after transfection with *wt*-hGH, using QIAGEN RNeasy kit (QIAGEN AG, Basel, Switzerland) including deoxyribonuclease treatment. Thereafter, total RNA was reverse transcribed (1 µg total RNA) in 25 µl reverse transcriptase reaction using oligo (deoxythymidine) 18 primers, 20 mM of each deoxynucleotide triphosphate, 10X first strand buffer solution, Moloney murine leukemia virus reverse transcriptase (Roche Molecular Biochemicals, Mannheim, Germany), and diethyl pyrocarbonate. The PCR mixture (25 µl total volume) consisted of primers (forward and reverse 900 nM) and the PCR universal master mix (PE Applied Biosystems). Negative controls included no template control and RNA control to check for genomic contamination. The primer pairs for rat GPR41, 43 were designed as described previously [12]. PCR conditions were as follows: 94°C for 5 min; 33 cycles at 94°C for 1 min, 58°C for 1 min 30 s, and 72°C for 40 s for GPR43 and GPR41; 94°C for 5 min; 25 cycles at 94°C for 30 s, 60°C for 45 s, and 72°C for 45 s for glyceraldehyde-3-phosphate dehydrogenase (GAPDH). Aliquots of the PCR reaction were analysed on 1.5% Metaphor gels (Bioconcept, Allschwil, Switzerland).

### 2.5. Western blot analysis

Cellular proteins were extracted 24 h after GHRH and/or butyrate treatment from GC-GHRHR cells using RIPA lysis buffer; 50 µg of total cell lysates were separated on 15% SDS-PAGE gel and blotted on Immobilon P transfer membranes (Millipore, Bedford, MA) using a Trans-Blot semidry apparatus (Bio-Rad Laboratories, Hercules, CA). Membranes were probed with polyclonal rabbit anti-human GH antibodies (1000x) (ICN Pharmaceuticals, Inc., Eschwege, Germany) and with monoclonal mouse anti-β-actin (1000x) (Sigma Aldrich, Buchs, SG, Switzerland). For secondary antibodies, anti-rabbit (DakoCytomation, Glostrup, Denmark) or anti-mouse immunoglobulins (Santa Cruz Biotechnology, Labforce AG, Switzerland) were used. Protein bands were visualized by enhanced chemiluminescence substrate reagent and exposed on ECL Plus films (Amersham Pharmacia Biotech, Dubendorf, Switzerland). β-actin was used as a control for equal loading. For quantification, protein bands were densitometrically measured using Quantity One software. Cellular proteins extracted from untransfected GC cells were used as a negative control.

### 2.6. Extracellular hGH secretion measurement after GHRH and/or butyrate treatment

Aliquots of culture medium were collected 24 h after 10 nM GHRH and/or 5 mM butyrate treatment. Extracellular hGH was measured in the aliquots of culture medium by the DSL-10-1900 *Active* hGH enzyme-linked immunoabsorbent assay (ELISA) kit as previously described [15]. The results of hGH secretion were normalized to a total protein content. The assay kit used was highly specific for the exclusive detection of hGH.

### 2.7. Dual luciferase reporter assay

GC-GHRHR cells were transiently transfected with the *Renilla* luciferase control vector and with the reporter construct pCREluc, a luciferase expression vector, under the control of 16 cAMP-responsive elements [16], using nucleofection as described above. 1.5 mM 8-Br-cAMP (Sigma Aldrich, Poole, UK) was used as a positive control. 20–24 h post-treatment transfected cells were lysed and assayed for dual luciferase activities as described by the manufacturer (Promega, Dübendorf, Switzerland).

### 2.8. siRNA-mediated gene silencing of GPR41 and 43 and quantitative real-time PCR analysis (SYBR Green)

GC-GHRHR cells were transiently transfected with rat GPR41 siRNA (5′-GGAGCUACGUGCUUCUCCU-3′) or rat GPR43 siRNA (5′-CUGCUAUUGGCGCUUUGUA-3′) (Sigma Aldrich, Buchs, Switzerland) using Amaxa nucleofection as described above. A mixture of four or more mismatches with known rat genes (siControl Non-Targeting siRNA Pool) (Pharmacon, Thermo Fisher Scientific, Wohlen, Switzerland) was used as a negative control to detect off-target effects. Two days after transfection, GPR41 and 43 mRNA expression were determined by quantitative real-time PCR (qRT-PCR) using the 7500 Fast Real-Time PCR System (Applied Biosystems, Foster City, CA, USA). In brief, PCR reactions were performed in 96-well plates (MicroAmp, Applied Biosystems) using cDNA prepared as described above. We used ABsolute QPCR SYBR Green Mix (ABgene, Thermo Fisher Scientific, Wohlen, Switzerland), 1 µl (20 pmol/µl) specific primers (Microsynth, Balgach, Switzerland) and 40 ng cDNA in a total volume of 25 µl. Relative expression values were determined by the comparative C_t_ method using 18S rRNA as the reference gene. Amplification curves and the mean Ct values were calculated using the 7500 Fast System SDS software (Applied Biosystems, LifeTechnologies, Basel, Switzerland).

48 h after silencing, cells were further re-transfected with *wt*-hGH and treated or not with 10 nM GHRH and/or 5 mM butyrate for 24 h. The extracellular GH secretion was measured as described in 2.6.

### 2.9. Intracellular calcium [Ca^2+^]_i_ measurements

GC-GHRHR cells, transiently transfected with *wt*-hGH and treated for 24 h with 5 mM butyrate, were plated in half-area clear bottom 96-well plates (Corning, CA, USA) at 75^3^ cells/well. 24 h later, following two washings with OPTIMEM medium +2.5 mM probenicid (Sigma-Aldrich, Buchs, Switzerland), cells were incubated with 2 µg/mL Fluo-4 AM (Invitrogen, LifeTechnologies, Basel, Switzerland) and Pluronic F-127 0.025% (w/v) (Invitrogen, LifeTechnologies, Basel, Switzerland) for 30 min in dark at 37°C. After washing twice with OPTIMEM medium, cell measurement was performed on a Synergy-4 instrument (BioTek, Highland Park, VT, USA) with an excitation band of 485/20 nm and fluorescence was measured at 528/20 nm. Baseline signal (*F*_0_) was recorded during 5 min before the addition of each stimulus. Subsequently, continuous fluorescence measurements were performed for 20 to 30 min. Ionomycin (Calbiochem, San Diego, CA, USA) stimulation was used as a positive control. Results are shown as *F*/*F*_0_ ratios after background subtraction, where *F* was the fluorescence signal intensity and *F*_0_ was the baseline as calculated by averaging the ten frames before stimulus application.

For [Ca^2+^]_i_ measurements in siRNA-treated cells, cells were further transfected with *wt*-hGH 48 h post-siRNA transfection and treated with 5 mM butyrate. After 24 h cells were seeded and treated as mentioned above.

### 2.10. Statistical analysis

Means and standard deviation of means were calculated for the 3 to 10 replications performed per experiment. For analysis of [Ca^2+^]_i_ measurements, peaks (maximum *F*/*F*_0_ ratio within 499 sec. after stimulus) were compared using Student's t-test, and means and slopes of *F*/*F*_0_ ratio in the plateau phase (≥500 sec. after stimulus) were compared using random coefficients mixed linear regression (random intercept and random slope per run, first-order serial autocorrelation) [17].

Specifically, the *lme* procedure of the *nlme* package in R 3.0.2 (R Foundation for Statistical Computing, Vienna, Austria) was used. In addition the area under the curves was calculated using trapezoidal rule and the results were compared using a Student's t-test.

For the remaining analyses, two-sided one–way ANOVA followed by Bonferroni's post hoc comparison tests was performed used GraphPad prism 5 (*p<0.05, **p<0.01, ***p<0.001). p-values <0.05 were considered significant.

## Results

### 3.1. Intracellular hGH production and hGH secretion after GHRH and/or butyrate treatment

**Figure 1:**
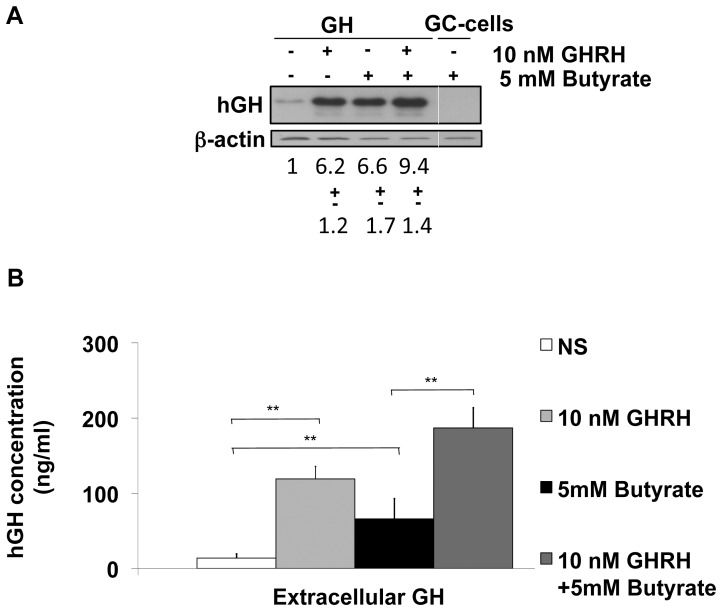
Analysis of intracellular hGH expression and extracellular hGH secretion after GHRH and/or butyrate treatment. (A) A representative Western Blot analysis of hGH is shown. The bands were densitometrically quantified and normalized to β-actin. The band corresponding to non-stimulated and non-treated conditions (NS) were set to 1 and all the remaining samples were compared accordingly. GC-cell non-transfected with *wt*-hGH were used as a negative control. (B) Measurements of extracellular hGH secretion. hGH secretion was measured in aliquots of culturing medium by DSL-GH ELISA and all the values are further normalized to total protein content. For all the experiments, the results represent a mean +/− SD of at least four independent quantification experiments. (*, p<0.05, **, p<0.01).

We assessed the impact of butyrate on intracellular hGH production by Western blot. After transfection with *wt*-hGH, GC-GHRHR cells were treated or not with 10 nM GHRH and/or 5 mM butyrate for 24 h. GHRH treatment evoked a 6.2 fold increase in hGH protein expression when compared to non-stimulated conditions. Treatment with butyrate induced a 6.6 fold increase while combined treatment with GHRH and butyrate induced a 9.4 fold increase in hGH protein expression over non-stimulated conditions (Fig. 1A).

We also measured the hGH secretion in the same experimental setting. Basal hGH release (NS) (mean: 14 ng/ml) significantly increased after GHRH treatment (mean: 119 ng/ml) (*p*<0.01). Butyrate treatment itself significantly increased hGH secretion (mean: 66 ng/ml) (*p*<0.01) compared to the basal level while in the presence of both GHRH and butyrate, extracellular GH secretion was significantly increased (mean: 187 ng/ml) (*p*<0.01) when compared to cells stimulated with butyrate only (Fig. 1B).

### 3.2. cAMP production in GC-GHRHR cells after GHRH and/or butyrate treatment

**Figure 2:**
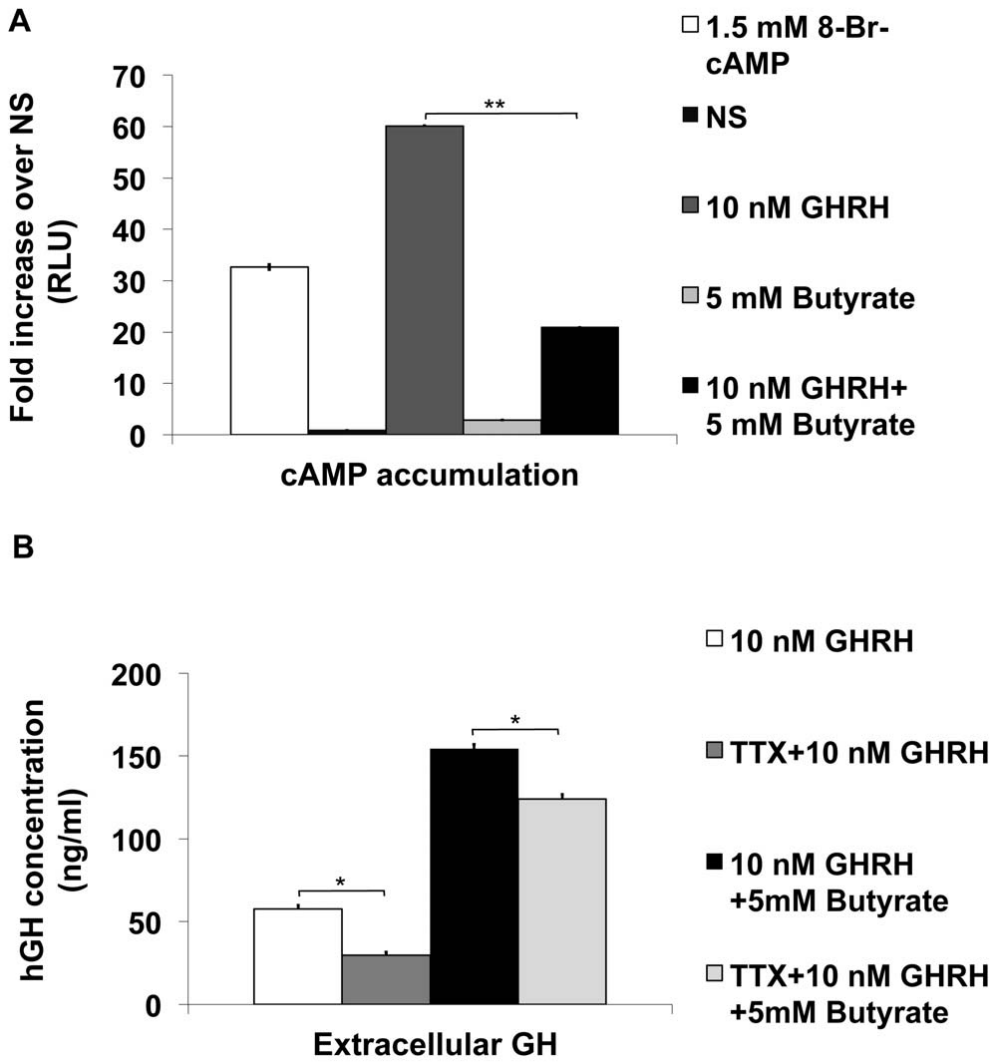
Involvement of cAMP and Na^+^ channels in butyrate-mediated hGH secretion. (A) Effect of butyrate on cAMP accumulation was assessed in GC-GHRHR cells co-transfected with a dual luciferase reporter vector and following 24 h incubation with 10 nM GHRH and/or 5 mM butyrate. Results are given as relative luciferase units (RLU) and expressed as fold increase over non-treated conditions (NS). (B) hGH secretion was measured in aliquots of culturing medium from GC-GHRHR cells transfected with *wt*-hGH after 24 h incubation with 10 nM GHRH and/or 5 mM butyrate and additional incubation with 30 µM tetrodotoxin (TTX). All the values are normalized to a total protein content. For all the experiments, the results represent a mean +/− SD of at four independent quantification experiments. (*, p<0.05, **, p<0.01).

Since butyrate increased intracellular hGH content and its extracellular release, we next investigated whether the main signalling pathway, the adenylate cyclase (AC)/cAMP/protein kinase (PKA) pathway, activated by GHRH, is activated in response to butyrate. A dual luciferase reporter assay showed that GHRH evoked an increase in cAMP levels through GHRHR activation, while butyrate itself induced no increase of cAMP levels compared to non-treated conditions (NS) (Fig. 2A). On the contrary, co-treatment with GHRH and butyrate decreased cAMP levels by 60% compared to those evoked by GHRH treatment alone. Our results indicate that the reduction in cAMP level induced by co-treatment with GHRH and butyrate may occur as a result of an interaction between different Gα subunits associated with either GPR41 (Gi/o inhibiting the cAMP production) or GHRHR (Gs catalysing the increase in cAMP levels), which are activated by butyrate or by GHRH.

### 3.3. GHRH- and/or butyrate-induced hGH secretion after Na^+^ channels blockade

Based on previous reports [18] demonstrating that GHRH depolarizes the cell membrane by activating Na^+^ channels resulting in the opening of calcium channels and a Ca^2+^ influx into the cells, we assessed the involvement of Na^+^ channels on butyrate-induced hGH release. hGH release was measured in response to GHRH and/or butyrate treatment with or without additional incubation with 30 µM tetrodotoxin, a selective Na^+^ channel blocker. In the presence of tetrodotoxin, we observed 20% reduction of GHRH-induced hGH secretion when compared to samples not treated with tetrodotoxin (Fig. 2B). Since a comparable reduction in hGH secretion was also observed after GHRH + butyrate stimulation, our results suggest that Na^+^ channels do not participate in the process of butyrate-induced hGH secretion.

### 3.4. GHRH and/or butyrate stimulation affect intracellular calcium [Ca^2+^]_i_ levels

**Figure 3:**
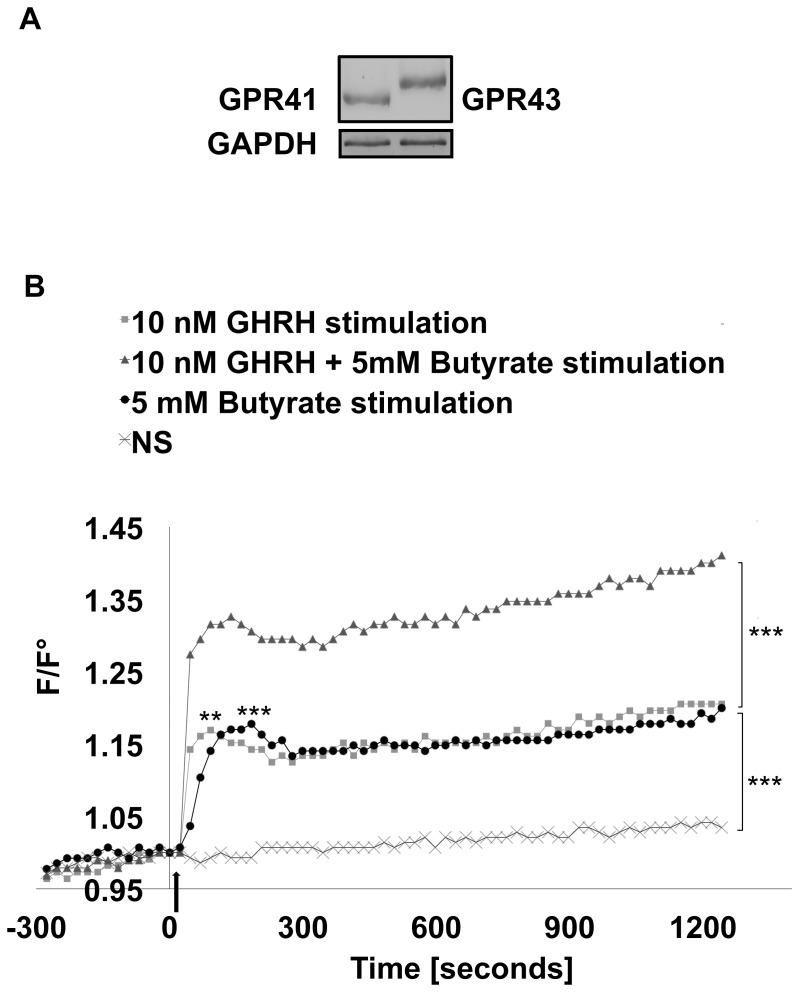
Intracellular calcium [Ca^2+^]_i_ response in GC-GHRHR cells mediated by GPR41 and 43 receptors. (A) GPR41 and GPR43 expression was detected by RT-PCR in GC-GHRHR cells. The expected sizes of the amplified DNA bands were 439 and 508 bp for GPR43 and GPR41, respectively. (B) Measurements of the [Ca^2+^]_i_ in GC-GHRHR cells, transiently transfected with *wt-*hGH and treated with butyrate, were assessed after stimulation with 10 nM GHRH and/or 5 mM butyrate. Addition of the stimulus is indicated with an arrow. One representative experiment of at least 5 independent experiments is shown (*, p<0.05, **, p<0.01, ***p<0.001).

Considering that butyrate might exert its action on somatotrophs through binding to the two putative SCFAs receptors, GPR41 and 43, we assessed their expression in GC-GHRHR cells transfected with *wt*-hGH by RT-PCR and found both receptors to be constitutively expressed (Fig 3A). The rise in [Ca^2+^]_i_ is a key event in any nutrient-induced GH secretion [19] and therefore butyrate might stimulate hGH release by enhancing [Ca^2+^]_I_ levels. To test this hypothesis, changes in [Ca^2+^]_i_ in GC-GHRHR cells expressing *wt*-hGH after stimulation with 10 nM GHRH and/or 5 mM butyrate were monitored from 20 to 30 minutes (Fig. 3B). In comparison to non-stimulated conditions (NS), GHRH stimulation induced a significant increase (p<0.001) in a biphasic Ca^2+^ oscillation with an initial sharp peak in [Ca^2+^]_I_, resulting from Ca^2+^ release from intracellular stores [19]–[21], followed by a moderate and long-lasting [Ca^2+^]_i_ rise due to the influx of Ca^2+^ through the opening of the Voltage Gated Calcium Channel (VGCCs) [20]. Interestingly, stimulation with butyrate led to a similar calcium response to that induced by GHRH (Fig. 3B). Combined stimulation with GHRH and butyrate induced a statistically significant biphasic Ca^2+^ increase (p<0.001), which was more pronounced in the plateau phase when compared to GHRH alone (Fig. 3B). To verify the specificity of the intracellular calcium response recorded, changes in [Ca^2+^]_i_ were also followed in the absence of stimuli (NS). Under the culture conditions employed, spontaneous Ca^2+^ oscillations were not observed, whereas a spontaneous transient rise in Ca^2+^ was only occasionally encountered. Finally, considering that extracellular acidosis induces Ca^2+^ oscillations [22], we confirmed that pH of the culture medium was not changed after the addition of butyrate (medium pH = 7.21, medium + sodium butyrate pH = 7.26, conditions: 37°C and 5% CO_2_).

### 3.5. Silencing of GPR41 and 43 by gene-specific siRNA's

**Figure 4:**
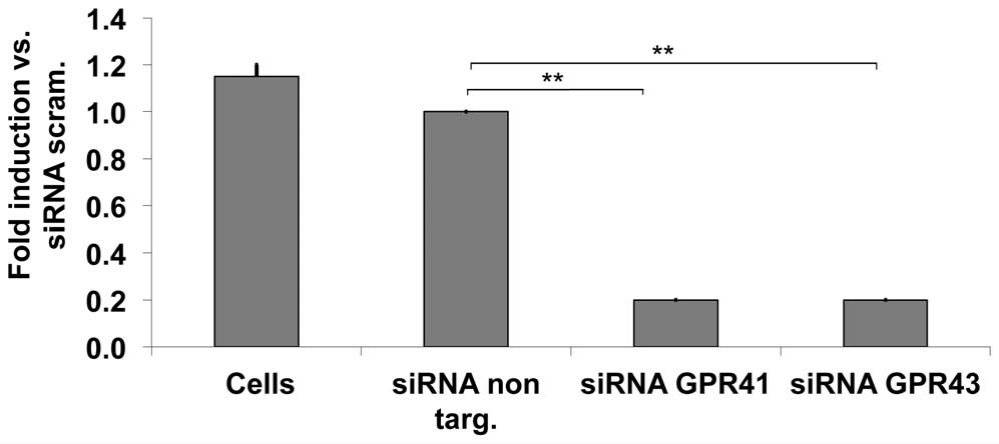
Silencing of GPR41 and GPR43 by gene-specific siRNAs detected by qRT-PCR (SYBR Green). GC-GHRHR cells were transiently transfected with either negative control (siRNA non targeting) or GPR41- or GPR43- specific siRNA and analysed after two days of incubation. Results are expressed as fold induction over siRNA non targeting condition. The results represent a mean +/− SD of four independent experiments. (*, p<0.05, **, p<0.01).

To further test the possible correlation between the expression of GPR41 and 43 and the increase in [Ca^2+^]_i_ in GC-GHRHR cells, we silenced GPR41 and 43 using gene-specific siRNA's. Silencing of rat GPR41 or GPR 43 expression using specific siRNA caused a significant decrease in the mRNA expression of both receptors when compared to the controls (cells transfected with siRNA non targeting) (Fig. 4). Two days after transfection, mRNA expression of GPR41 and 43 declined to less than 25% of that detected in control cells. Transfection of GC-GHRHR cells with siRNA non-targeting had no effect on the expression of GPR41 and GPR43 mRNA when compared to untransfected cells.

### 3.6. Effect of GHRH and/or butyrate stimulation on the [Ca^2+^]_I_ and extracellular hGH secretion after silencing of the GPR41 and 43 receptors

Further we wanted to test whether the silencing of GPR41 and 43 has a direct impact on [Ca^2+^]_i_ changes evoked by butyrate and/or GHRH and whether it directly affects hGH secretion, which would demonstrate a direct link between butyrate activation of GPR 41 and 43, elevation of [Ca^2+^]_I_ and enhanced hGH secretion. Therefore, two days after silencing, cells were re-transfected with *wt*-hGH, treated with butyrate and further incubated for 24 h when the [Ca^2+^]_i_ response to GHRH and/or butyrate stimulation was assessed.

**Figure 5:**
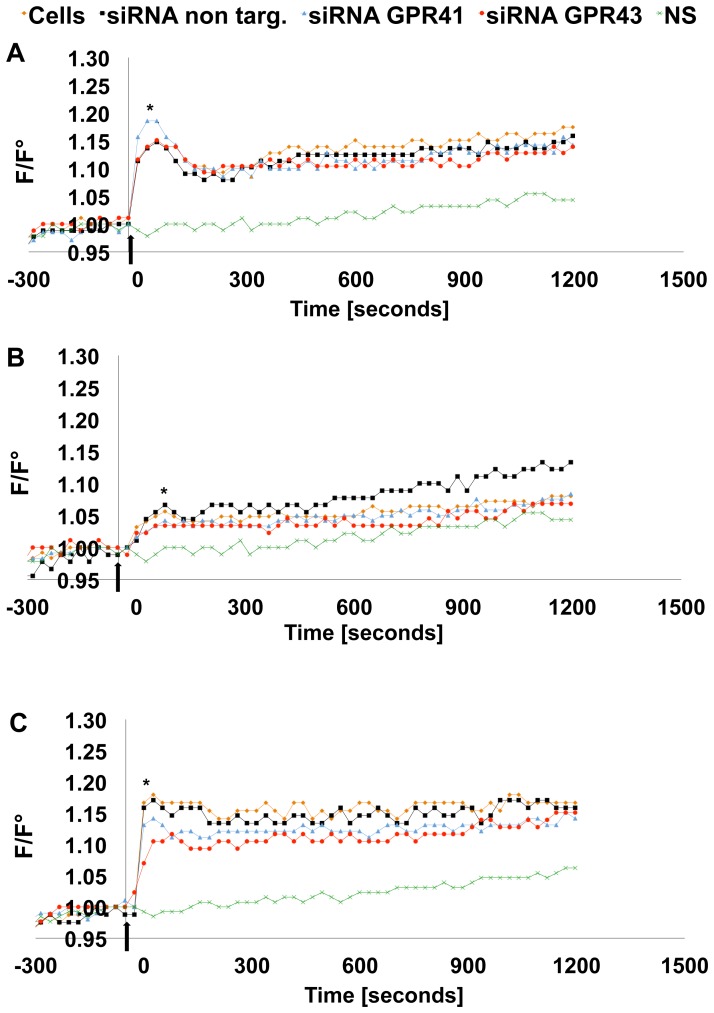
Intracellular calcium [Ca^2+^]_i_ response after GPR41 and 43 silencing. (A) [Ca^2+^]_i_ changes were measured in response to 10 nM GHRH or (B) in response to 5 mM butyrate and (C) in response to 10 nM GHRH +5 mM butyrate in GC-GHRHR cells transfected with *wt*-hGH and cells further expressing siRNA control (siRNA non targeting), GPR43-specific siRNA, or GPR41-specific siRNA previously treated for 24 h with 5 mM butyrate. Addition of the stimulus is indicated with an arrow. Mean values and area under the peaks were compared for statistics. One representative experiment of at least 5 independent experiments is shown (*, p<0.05, **, p<0.01).

In cells transfected with siRNA non targeting the [Ca^2+^]_i_ response evoked by butyrate and/or GHRH was statistically not different from that measured in GC-GHRHR cells (Fig. 5A–C).

In presence of GHRH (Fig. 5A), silencing of GPR41 induced a [Ca^2+^]_i_ response higher in the peak phase (p<0.05) compared to the control; while, after silencing of GPR43, the [Ca^2+^]_i_ response following GHRH stimulation did not change. On the other hand, the [Ca^2+^]_i_ response to butyrate stimulation was lower in the peak phase (p<0.05) following silencing of GPR41 or 43 (Fig. 5B). Co-stimulation with butyrate and GHRH (Fig. 5C) showed a decreased [Ca^2+^]_i_ response (p<0.05), in the peak phase, following silencing of GPR41 or 43.

**Figure 6:**
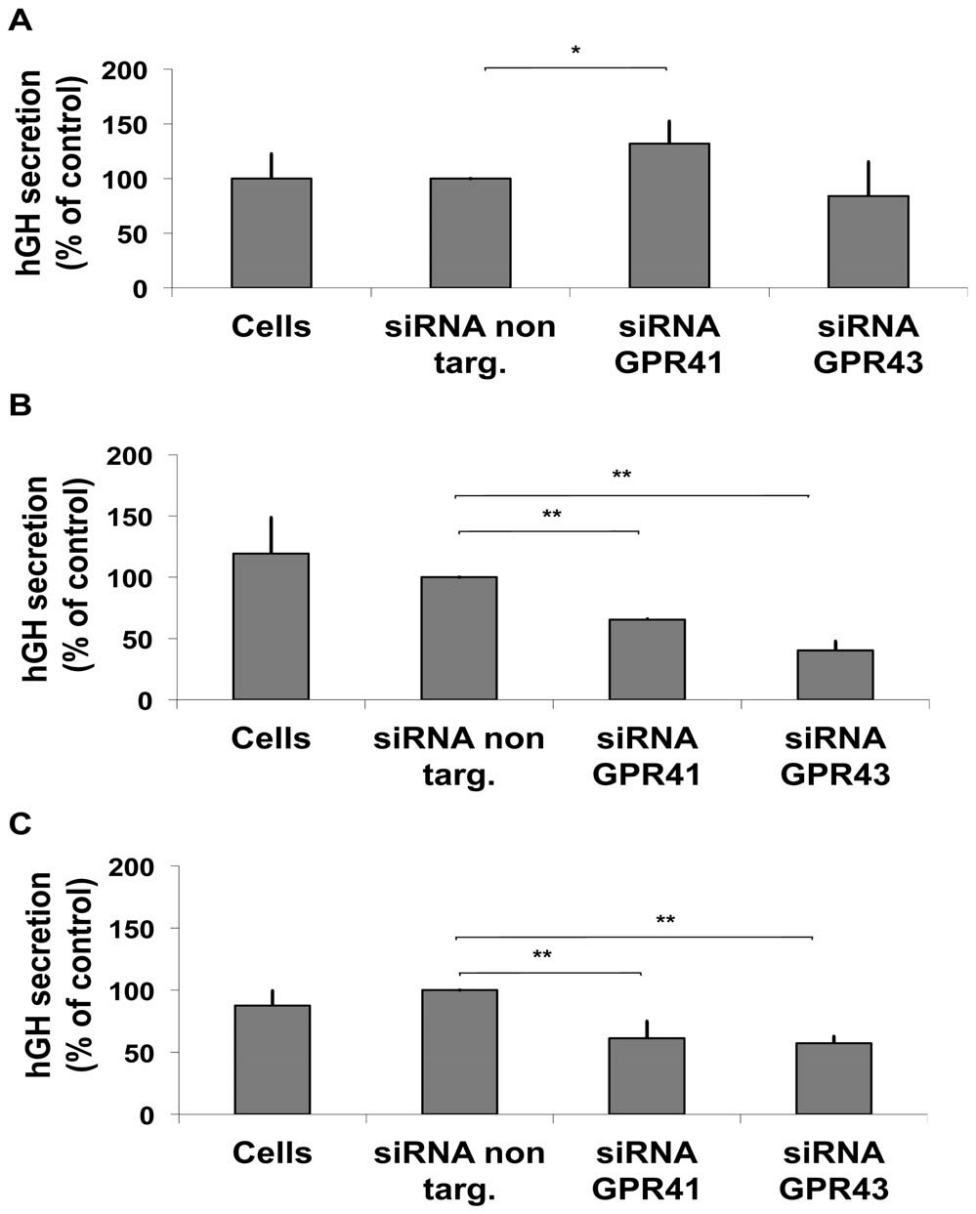
Extracellular hGH secretion after GPR41 and GPR43 silencing. hGH secretion was measured by DSL-GH ELISA in aliquots of culturing medium from GC-GHRHR cells transfected with either negative control (siRNA non targeting) or GPR41- or GPR43- specific siRNAs. After 48 h of silencing, cells were re-transfected with *wt*-hGH and cultured for additional 24 h with 10 nM GHRH (A), 5 mM butyrate (B) and 5 mM butyrate plus 10 nM GHRH (C). All the values are expressed as percentage increase over siRNA non targeting condition. The results represent a mean +/− SD of four independent experiments (*, p<0.05, **, p<0.01).

Finally, we analysed the impact of GPR41 and 43 silencing on extracellular hGH secretion. The silencing of GPR41 evoked a significant increase (p<0.05) in GHRH-induced hGH secretion compared to cells transfected with siRNA non targeting (p<0.05), while silencing of GPR43 had no impact on GHRH-induced hGH secretion (Fig 6A). Furthermore, butyrate-induced hGH secretion was significantly decreased (p<0.01) after the silencing of GPR41 or GPR43 when compared to cells transfected with siRNA non-targeting (Fig 6B). In the presence of GHRH and butyrate (Fig. 6C), GPR43 or GPR41 silencing caused a significant decrease in hGH secretion when compared to the controls (p<0.01).

## Discussion

The aim of this study was to test the impact of butyrate on hGH secretion and production in a rat pituitary tumour cell line and to investigate a potential role for SCFAs' receptors, GPR41 and GPR43, in mediating the effect of butyrate on [Ca^2+^]_I_ and hGH secretion.

We showed that in rat pituitary tumour cells, butyrate enhances hGH release in both not-stimulated and GHRH-stimulated conditions through the activation of GPR41 and 43 receptors.

Reported effects of butyrate on GH secretion obtained *in vitro* are still controversial, since it has been reported that butyrate inhibits GH synthesis in GH1 cells [23] but stimulates GH synthesis in GH3 [24] and GH4Cl cells [25]. More recently *Ishiwata et al.* [12] demonstrated a suppressive effect of butyrate on rat GH secretion in a primary culture of rat anterior pituitary cells, which is however a heterogeneous cell population consisting of five different types of neuroendocrine cells. Due to its short plasma half-life [26], [27], *in vivo* testing of the effects of butyrate may be difficult. Infused butyrate is rapidly metabolised and the plasma concentration is well below the concentrations in the mM range that are generally needed to produce effects *in vitro*. However, several compounds structurally related to butyrate, like gamma-hydroxybutyrate (GHB or sodium oxylibate) [28], [29], beta-hydroxy-beta methylbutyrate (HMβ) or even the infusion of BHB alone have been shown to significantly increase GH secretion in humans [30].

Efforts to determine the precise mechanism responsible for this response have been confounded by the fact that butyrate can act on both the hypothalamus and pituitary [12], [31]. So far it has been suggested that butyrate-induced GHRH release is sufficient to elicit GH secretion but no studies have provided convincing data to support this hypothesis. Therefore, based on current data, it remains unclear whether the potentiated GH secretion responses arise from the direct action of butyrate on the somatotroph cells.

In our cell model, we used GC cells, a homogeneous neuroendocrine cell population that produce endogenous rGH but do not express rat GHRHR and are thus refractory to GHRH stimulation. We could thus transfect them with *wt*-hGH and stimulate hGH production and secretion with GHRH (Robinson, I. C., unpublished data).

In the somatotroph cells of the pituitary gland, the release of GH is primarily regulated by GHRH and the underlying mechanism involves an increase in cAMP level and changes in [Ca^2+^]_I_ [32], [33]. Our results show that in spite of having no effect on cAMP levels and a reducing effect on GHRH-stimulated cAMP accumulation, butyrate is still able to stimulate GH secretion. This suggests that its enhancing effect on GH release is independent of its effect on cAMP accumulation and is mediated by a mechanism involving changes in [Ca^2+^]_i_. This notion is based on observations that [Ca^2+^]_i_ response to butyrate stimulation was of similar magnitude to that evoked by GHRH. In both cases, [Ca^2+^]_i_ displayed either a peak phase resulting from Ca^2+^ release from intracellular stores, or a plateau type phase characterized by a moderate and long-lasting [Ca^2+^]_I_, rise due to the influx of Ca^2+^ through the opening of Voltage Gated Calcium Channel (VGCCs) [20], [21], [34]. A Ca^2+^-dependent, cAMP-independent action of butyrate raises the possibility that butyrate may act on a receptor distinct from GHRHR.

Based on our hypothesis that butyrate increases [Ca^2+^]_i_ through activation of GPR41 and 43 and to further demonstrate their involvement in butyrate-induced increase of hGH secretion, we silenced both receptors by gene-specific siRNA's.

GC-GHRHR cells were transfected with rat GPR41 or GPR43 siRNA, which largely reduced the GPR41 or 43 mRNA expression. Silencing of GPR41 or GPR43 significantly reduced the [Ca^2+^]_i_ response induced by butyrate stimulation or by the co-stimulation with butyrate and GHRH to the same extent. Both receptors may in fact induce an increase in [Ca^2+^]_i_ via inositol 1,4,5-trisphosphate formation [7]. The [Ca^2+^]_i_ reduction following downregulation of GPR41 or 43 was unlikely to be due to non-specific silencing of key components involved in Ca^2+^ signalling, since Ca^2+^ signals induced by ionomycin (Ca^2+^ ionophore) remained unaffected by GPR41 or 43 –specific siRNA silencing.

Moreover silencing of GPR41 or 43 largely abolished GH secretion induced by butyrate or co-stimulation with butyrate and GHRH. The inhibition of GH secretion parallels the inhibitory action of GPR41 or 43 silencing on the Ca^2+^ level. Whether other mechanisms, which are known to be coupled to GPR43 activation such as activation of protein kinase C [35], act in concert with or independently from the rise in Ca^2+^ to stimulate GH release, remains to be determined. Considering that a rise in [Ca^2+^]_i_ is a key event in triggering GH exocytosis [32], [33], [36], a causal relation between the two events seems plausible. Together, these results indicate that the butyrate-induced [Ca^2+^]_I_ increase and the corresponding increase in hGH secretion is mainly mediated by activation of GPR41 and 43.

On the other hand, silencing of GPR41 but not of GPR43 induced a significant increase in GHRH-induced [Ca^2+^]_i_ and GH secretion, which could be explained by the fact that GPR41 is a Gi/o-coupled GPCR related to the inhibition of cAMP production [7], [9]. Our results suggest that GHRH-induced reduction in cAMP level is likely due to the interaction in post receptor signalling between the two different Gα subunits of GPR41 or 43 and GHRHR. Moreover, in our cell model, butyrate acts synergistically with GHRH in its effect on GH release and this action could be mediated by GPR43 as they share the same pathway (Gβγ-PLC-PKC).

Butyrate is considered a minor nutrient source produced by bacteria in the gut. It was recently shown that GPR41 and 43, for which butyrate is one of the physiologically endogenous ligands, provide an additional function for this molecule as an initiating element in signalling cascade. In addition, it was shown that butyrate stimulates leptin production in adipocytes through the activation of GPR41 [37]. Leptin stimulates GH secretion in rodents at the level of the hypothalamus by regulating GHRH and SRIF activity [38], [39].

GPR41 and 43 are expressed not only in the intestine, but also in the immune system and sympathetic nervous system where they regulate energy metabolism [7], [9], [11], [37], [40]. Plasma concentrations of butyrate and ß-hydroxybutyrate (BHB) are normally in the range of 50–100 µM [41]. During short-term food deprivation (24 h–48 h), levels gradually increase to low millimolar plasma concentrations and exceed 10 mM during starvation ketoacidosis or adherence to a ketogenic diet [42], [43], attaining a sufficient concentration to activate the receptors on other tissues not directly exposed to the intestinal lumen. The metabolic response to fasting involves an increase in circulating levels of GH and free fatty acids and resistance to insulin's actions on glucose metabolism. Endogenous GH secretion rates are enhanced 5-fold by a 2-day fast in healthy young men [44], secondary to fasting-associated reduction in IGF-1 concentration and the consequent loss of feedback inhibition. However, pulsatile GH secretion increases before a significant reduction in total serum IGF-1 concentration occurs [45] and the role of GH is to change energy substrate utilization by liberating free fatty acids [1].

In conclusion, we find that butyrate stimulates GH secretion through activation of receptors different to those used by GHRH, (the “classical” inducer of the GH release in physiology). This highlights the possibility that butyrate acts as a potential metabolic intermediary, which may contribute to the metabolic actions of GH during fasting. Finally, these findings suggest a role of butyrate in the regulation of GH axis function, possibly in combination with other factors such as acetate and ghrelin.

## References

[1] Norrelund H (2005) The metabolic role of growth hormone in humans with particular reference to fasting. Growth Horm IGF Res 15: 95–122. 15809014 10.1016/j.ghir.2005.02.005

[2] Bratusch-Marrain PR, Smith D, DeFronzo RA (1982) The effect of growth hormone on glucose metabolism and insulin secretion in man. J Clin Endocrinol Metab 55: 973–982. 6749883 10.1210/jcem-55-5-973

[3] Rizza RA, Mandarino LJ, Gerich JE (1982) Effects of growth hormone on insulin action in man. Mechanisms of insulin resistance, impaired suppression of glucose production, and impaired stimulation of glucose utilization. Diabetes 31: 663–669. 6761205 10.2337/diab.31.8.663

[4] Copeland KC, Nair KS (1994) Acute growth hormone effects on amino acid and lipid metabolism. J Clin Endocrinol Metab 78: 1040–1047. 8175957 10.1210/jcem.78.5.8175957

[5] Shimazu T, Hirschey MD, Newman J, He W, Shirakawa K, et al (2013) Suppression of oxidative stress by beta-hydroxybutyrate, an endogenous histone deacetylase inhibitor. Science 339: 211–214. 23223453 10.1126/science.1227166 PMC3735349

[6] Cahill GF Jr (2006) Fuel metabolism in starvation. Annu Rev Nutr 26: 1–22. 16848698 10.1146/annurev.nutr.26.061505.111258

[7] Le Poul E, Loison C, Struyf S, Springael JY, Lannoy V, et al (2003) Functional characterization of human receptors for short chain fatty acids and their role in polymorphonuclear cell activation. J Biol Chem 278: 25481–25489. 12711604 10.1074/jbc.M301403200

[8] Juul A, Jorgensen JOL (2000) Growth Hormone in Adults: Physiological and Clinical Aspects: Cambridge University Press. 498 p.

[9] Brown AJ, Goldsworthy SM, Barnes AA, Eilert MM, Tcheang L, et al (2003) The Orphan G protein-coupled receptors GPR41 and GPR43 are activated by propionate and other short chain carboxylic acids. J Biol Chem 278: 11312–11319. 12496283 10.1074/jbc.M211609200

[10] Wang A, Gu Z, Heid B, Akers RM, Jiang H (2009) Identification and characterization of the bovine G protein-coupled receptor GPR41 and GPR43 genes. J Dairy Sci 92: 2696–2705. 19448003 10.3168/jds.2009-2037

[11] Nilsson NE, Kotarsky K, Owman C, Olde B (2003) Identification of a free fatty acid receptor, FFA2R, expressed on leukocytes and activated by short-chain fatty acids. Biochem Biophys Res Commun 303: 1047–1052. 12684041 10.1016/s0006-291x(03)00488-1

[12] Ishiwata H, Katoh K, Chen C, Yonezawa T, Sasaki Y, et al (2005) Suppressing actions of butyrate on growth hormone (GH) secretion induced by GH-releasing hormone in rat anterior pituitary cells. Gen Comp Endocrinol 143: 222–230. 15927184 10.1016/j.ygcen.2005.03.015

[13] Bancroft FC (1973) Intracellular location of newly synthesized growth hormone. Exp Cell Res 79: 275–278. 4205699 10.1016/0014-4827(73)90445-x

[14] Petkovic V, Godi M, Lochmatter D, Eble A, Fluck CE, et al (2010) Growth hormone (GH)-releasing hormone increases the expression of the dominant-negative GH isoform in cases of isolated GH deficiency due to GH splice-site mutations. Endocrinology 151: 2650–2658. 20351314 10.1210/en.2009-1280

[15] Besson A, Salemi S, Deladoey J, Vuissoz JM, Eble A, et al (2005) Short stature caused by a biologically inactive mutant growth hormone (GH-C53S). J Clin Endocrinol Metab 90: 2493–2499. 15713716 10.1210/jc.2004-1838

[16] Fluck CE, Martens JW, Conte FA, Miller WL (2002) Clinical, genetic, and functional characterization of adrenocorticotropin receptor mutations using a novel receptor assay. J Clin Endocrinol Metab 87: 4318–4323. 12213892 10.1210/jc.2002-020501

[17] Everitt B, Rabe-Hesketh S (2001) Analyzing Medical Data Using S-PLUS: Springer. 486 p.

[18] Yang SK, Wang K, Parkington H, Chen C (2008) Involvement of tetrodotoxin-resistant Na+ current and protein kinase C in the action of growth hormone (GH)-releasing hormone on primary cultured somatotropes from GH-green fluorescent protein transgenic mice. Endocrinology 149: 4726–4735. 18535104 10.1210/en.2008-0405

[19] Chang JP, Habibi HR, Yu Y, Moussavi M, Grey CL, et al (2012) Calcium and other signalling pathways in neuroendocrine regulation of somatotroph functions. Cell Calcium 51: 240–252. 22137240 10.1016/j.ceca.2011.11.001

[20] Chen C, Xu R, Clarke IJ, Ruan M, Loneragan K, et al (2000) Diverse intracellular signalling systems used by growth hormone-releasing hormone in regulating voltage-gated Ca2+ or K channels in pituitary somatotropes. Immunol Cell Biol 78: 356–368. 10947860 10.1046/j.1440-1711.2000.00917.x

[21] Yang SK, Steyn F, Chen C (2012) Influence of membrane ion channel in pituitary somatotrophs by hypothalamic regulators. Cell Calcium 51: 231–239. 22243899 10.1016/j.ceca.2011.12.005

[22] Nakamura K, Kamouchi M, Arimura K, Nishimura A, Kuroda J, et al (2012) Extracellular acidification activates cAMP responsive element binding protein via Na+/H+ exchanger isoform 1-mediated Ca(2)(+) oscillation in central nervous system pericytes. Arterioscler Thromb Vasc Biol 32: 2670–2677. 22922957 10.1161/ATVBAHA.112.254946

[23] Stanley F, Samuels HH (1984) n-Butyrate effects thyroid hormone stimulation of prolactin production and mRNA levels in GH1 cells. J Biol Chem 259: 9768–9775. 6086648

[24] Yen PM, Tashjian AH Jr (1981) Short chain fatty acids increase prolactin and growth hormone production and alter cell morphology in the GH3 strain of rat pituitary cells. Endocrinology 109: 17–22. 7238401 10.1210/endo-109-1-17

[25] Dannies PS, Gautvik KM, Tashjian AH Jr (1976) A possible role of cyclic AMP in mediating the effects of thyrotropin-releasing hormone on prolactin release and on prolactin and growth hormone synthesis in pituitary cells in culture. Endocrinology 98: 1147–1159. 177274 10.1210/endo-98-5-1147

[26] Miller AA, Kurschel E, Osieka R, Schmidt CG (1987) Clinical pharmacology of sodium butyrate in patients with acute leukemia. Eur J Cancer Clin Oncol 23: 1283–1287. 3678322 10.1016/0277-5379(87)90109-x

[27] Chen ZX, Breitman TR (1994) Tributyrin: a prodrug of butyric acid for potential clinical application in differentiation therapy. Cancer Res 54: 3494–3499. 8012972

[28] Vescovi PP, Coiro V (2001) Different control of GH secretion by gamma-amino- and gamma-hydroxy-butyric acid in 4-year abstinent alcoholics. Drug Alcohol Depend 61: 217–221. 11164685 10.1016/s0376-8716(00)00149-6

[29] Volpi R, Chiodera P, Caffarra P, Scaglioni A, Malvezzi L, et al (2000) Muscarinic cholinergic mediation of the GH response to gamma-hydroxybutyric acid: neuroendocrine evidence in normal and parkinsonian subjects. Psychoneuroendocrinology 25: 179–185. 10674281 10.1016/s0306-4530(99)00048-7

[30] Quabbe HJ, Trompke M, Luyckx AS (1983) Influence of ketone body infusion on plasma growth hormone and glucagon in man. J Clin Endocrinol Metab 57: 613–618. 6348066 10.1210/jcem-57-3-613

[31] Yudkoff M, Daikhin Y, Nissim I, Horyn O, Lazarow A, et al (2005) Response of brain amino acid metabolism to ketosis. Neurochem Int 47: 119–128. 15888376 10.1016/j.neuint.2005.04.014

[32] Lussier BT, French MB, Moore BC, Kraicer J (1991) Free intracellular Ca2+ concentration ([Ca2+]i) and growth hormone release from purified rat somatotrophs. I. GH-releasing factor-induced Ca2+ influx raises [Ca2+]i. Endocrinology 128: 570–582. 1846113 10.1210/endo-128-1-570

[33] Lussier BT, French MB, Moor BC, Kraicer J (1991) Free intracellular Ca2+ concentration and growth hormone (GH) release from purified rat somatotrophs. III. Mechanism of action of GH-releasing factor and somatostatin. Endocrinology 128: 592–603. 1670926 10.1210/endo-128-1-592

[34] Sun S, Li W, Zhang H, Zha L, Xue Y, et al (2012) Requirement for store-operated calcium entry in sodium butyrate-induced apoptosis in human colon cancer cells. Biosci Rep 32: 83–90. 21699495 10.1042/BSR20110062

[35] Kimura I, Ozawa K, Inoue D, Imamura T, Kimura K, et al (2013) The gut microbiota suppresses insulin-mediated fat accumulation via the short-chain fatty acid receptor GPR43. Nat Commun 4: 1829. 23652017 10.1038/ncomms2852 PMC3674247

[36] Lussier BT, Wood DA, French MB, Moor BC, Kraicer J (1991) Free intracellular Ca2+ concentration ([Ca2+]i) and growth hormone release from purified rat somatotrophs. II. Somatostatin lowers [Ca2+]i by inhibiting Ca2+ influx. Endocrinology 128: 583–591. 1670925 10.1210/endo-128-1-583

[37] Xiong Y, Miyamoto N, Shibata K, Valasek MA, Motoike T, et al (2004) Short-chain fatty acids stimulate leptin production in adipocytes through the G protein-coupled receptor GPR41. Proc Natl Acad Sci U S A 101: 1045–1050. 14722361 10.1073/pnas.2637002100 PMC327148

[38] Carro E, Senaris R, Considine RV, Casanueva FF, Dieguez C (1997) Regulation of in vivo growth hormone secretion by leptin. Endocrinology 138: 2203–2206. 9112421 10.1210/endo.138.5.5238

[39] Tannenbaum GS, Gurd W, Lapointe M (1998) Leptin is a potent stimulator of spontaneous pulsatile growth hormone (GH) secretion and the GH response to GH-releasing hormone. Endocrinology 139: 3871–3875. 9724042 10.1210/endo.139.9.6206

[40] Nohr MK, Pedersen MH, Gille A, Egerod KL, Engelstoft MS, et al (2013) GPR41/FFAR3 and GPR43/FFAR2 as cosensors for short-chain fatty acids in enteroendocrine cells vs FFAR3 in enteric neurons and FFAR2 in enteric leukocytes. Endocrinology 154: 3552–3564. 23885020 10.1210/en.2013-1142

[41] Cummings BP, Bettaieb A, Graham JL, Stanhope KL, Dill R, et al (2011) Subcutaneous administration of leptin normalizes fasting plasma glucose in obese type 2 diabetic UCD-T2DM rats. Proc Natl Acad Sci U S A 108: 14670–14675. 21873226 10.1073/pnas.1107163108 PMC3167517

[42] Fulop M, Murthy V, Michilli A, Nalamati J, Qian Q, et al (1999) Serum beta-hydroxybutyrate measurement in patients with uncontrolled diabetes mellitus. Arch Intern Med 159: 381–384. 10030312 10.1001/archinte.159.4.381

[43] Gilbert DL, Pyzik PL, Freeman JM (2000) The ketogenic diet: seizure control correlates better with serum beta-hydroxybutyrate than with urine ketones. J Child Neurol 15: 787–790. 11198492 10.1177/088307380001501203

[44] Hartman ML, Veldhuis JD, Johnson ML, Lee MM, Alberti KG, et al (1992) Augmented growth hormone (GH) secretory burst frequency and amplitude mediate enhanced GH secretion during a two-day fast in normal men. J Clin Endocrinol Metab 74: 757–765. 1548337 10.1210/jcem.74.4.1548337

[45] Ho KY, Veldhuis JD, Johnson ML, Furlanetto R, Evans WS, et al (1988) Fasting enhances growth hormone secretion and amplifies the complex rhythms of growth hormone secretion in man. J Clin Invest 81: 968–975. 3127426 10.1172/JCI113450 PMC329619

